# Global Access to Safe Water: Accounting for Water Quality and the Resulting Impact on MDG Progress

**DOI:** 10.3390/ijerph9030880

**Published:** 2012-03-14

**Authors:** Kyle Onda, Joe LoBuglio, Jamie Bartram

**Affiliations:** The Water Institute, Gillings School of Global Public Health, University of North Carolina at Chapel Hill, 135 Dauer Drive, CB #7431, Chapel Hill, NC 27599, USA; Email: konda@live.unc.edu (K.O.); lobuglio@unc.edu (J.L.)

**Keywords:** water quality, drinking water, water safety, Millennium Development Goals (MDGs), Joint Monitoring Programme (JMP)

## Abstract

Monitoring of progress towards the Millennium Development Goal (MDG) drinking water target relies on classification of water sources as “improved” or “unimproved” as an indicator for water safety. We adjust the current Joint Monitoring Programme (JMP) estimate by accounting for microbial water quality and sanitary risk using the only-nationally representative water quality data currently available, that from the WHO and UNICEF “Rapid Assessment of Drinking Water Quality”. A principal components analysis (PCA) of national environmental and development indicators was used to create models that predicted, for most countries, the proportions of piped and of other-improved water supplies that are faecally contaminated; and of these sources, the proportions that lack basic sanitary protection against contamination. We estimate that 1.8 billion people (28% of the global population) used unsafe water in 2010. The 2010 JMP estimate is that 783 million people (11%) use unimproved sources. Our estimates revise the 1990 baseline from 23% to 37%, and the target from 12% to 18%, resulting in a shortfall of 10% of the global population towards the MDG target in 2010. In contrast, using the indicator “use of an improved source” suggests that the MDG target for drinking-water has already been achieved. We estimate that an additional 1.2 billion (18%) use water from sources or systems with significant sanitary risks. While our estimate is imprecise, the magnitude of the estimate and the health and development implications suggest that greater attention is needed to better understand and manage drinking water safety.

## 1. Introduction

The United Nations Millennium Development Goals (MDGs) include Target 7c, to halve the “proportion of the population without sustainable access to safe drinking-water” between 1990 and 2015 [1]. The Joint Monitoring Programme for Water Supply and Sanitation (JMP) of the World Health Organization (WHO) and the United Nations Children’s Fund (UNICEF) reports progress towards meeting this goal [2]. The corresponding MDG indicator is the “proportion of households using water from an improved source,” and is reported on a country-by-country basis [1]. Sources are classified as improved or unimproved as shown in Table 1, according to whether they are “protected from outside contamination” [2]. The MDG indicator thereby conflates access to certain water sources with use of safe water. However, data and monitoring mechanisms regarding the safety of water sources at a national scale when the MDG targets were cast were, and remain, scant. As such, at the time the MDGs were developed, there was no credible alternative approach to an indicator that allows for the calculation of a percentage figure easily aggregated to the country and global scales and amenable for use as a target given the types of data available.

**Table 1:** JMP Classification of drinking-water source types as improved or unimproved [2].

Source class	Type of source
Unimproved drinking-water source	Unprotected dug well, unprotected spring, cart with small tank or drum, surface water (e.g., river, dam, lake, pond, stream, canal or irrigation channel) and bottled water
Improved drinking-water source (piped to dwelling, plot or yard)	Piped water connection located inside the user’s dwelling, plot or yard
Improved drinking-water source (other sources)	Public taps or standpipes, tube wells or boreholes, protected dug wells, protected springs and rainwater collection

Using this approach, WHO and UNICEF estimate that 5.8 billion people used improved sources in 2010, with 783 million using unimproved water sources [3]. Treating use of an improved source as an indicator for use of safe water is likely to overestimate the population using safe water, since some improved sources may provide water that is microbiologically or chemically contaminated whether at source or by the time it reaches the home and is consumed [4,5]. On the other hand, most unimproved sources do not provide safe drinking water, so under-accounting of safe water coverage due to unimproved sources providing safe water is likely to be small [6,7].

In 2010, WHO and UNICEF released data on water quality and sanitary risk (*i.e.*, risk of contamination) associated with improved sources from five countries as part of the Rapid Assessment of Drinking-Water Quality (RADWQ) study which had been undertaken between October 2004 and April 2005 [8,9,10,11,12]. The RADWQ study is the only source of nationally-representative drinking water quality data amenable to analysis for microbial contamination disaggregated by water source type. A recent analysis of these data found that accounting for water source compliance with WHO water quality guidelines significantly reduced the estimates of safe water access relative to the JMP figures, as well as national-level progress towards the MDG target, in four of the five countries [13]. However, this study did not account for sanitary risk, and did not extrapolate beyond the five countries for which data were available. We use both the water quality and sanitary risk data from the RADWQ studies to extrapolate to other countries, and estimate global figures. We believe our study is the first to use the available nationally-representative water safety data to estimate the proportion of the global population with access to safe drinking water and the concomitant impact on estimates of global progress towards MDG Target 7c with a method that accounts for differences between countries in factors affecting water source safety.

## 2. Methods

### 2.1. Drinking Water Source Type Classification

Here we considered “piped” sources as the subset of MDG-classified improved sources that are piped connections to a user’s dwelling, plot, or yard, and “other-improved” sources as all other improved drinking water sources as described in Table 1.

### 2.2. Drinking Water Quality Data

The RADWQ data was collected following the methodology described in Howard *et al.* [14]. The RADWQ data includes a nationally-representative sample of approximately 1,600 improved water sources in each of Ethiopia, Jordan, Nicaragua, Nigeria, and Tajikistan [8,9,10,11,12]. A population-weighted sample was taken of each source type that provided water to at least 5% of the national population in four of the five countries [8,9,10,11,12]. In Nicaragua, no single improved source type other than utility piped supplies covered more than 5% of the population, so several other-improved source types, each covering less than 5% of the population, were sampled [11]. Each sampled source was tested for thermotolerant coliform bacteria (TTC), fluoride, arsenic, and nitrate, and subjected to a standardized sanitary inspection [14]. Sanitary inspections in the RADWQ surveys identified risk factors for faecal contamination for a water source from a standardized list for each water source type of 10 common risks [8,9,10,11,12]. Examples of sanitary risks on these lists included pipe breaks, supply discontinuities, poor drainage, and proximity to latrines and animal waste. The RADWQ studies reported sanitary risk as a categorical variable with four levels: 0–, 3–5, 6–8, and 9–10 sanitary risks [8,9,10,11,12]. The RADWQ studies also provided cross-tabulations of sanitary risk levels and TTC contamination levels, except in the case of Nicaragua, which only reported the percentage of contaminated water sources in each sanitary risk level [8,9,10,11,12].

For each of the five countries, we used the RADWQ data to compute the percentages of piped water sources and other-improved water sources that were “safe” by virtue of not testing positive for TCC [15]. We computed the percentages of safe sampled piped and other-improved sources that also had greater than two sanitary risks on the sanitary risk inspection that accompanied the water quality tests for each sampled water source. We used this threshold to identify systems with all but the lowest aggregated level of sanitary risk reported in the RADWQ studies. We considered any one sanitary risk to represent an elevated risk of water contamination or re-contamination that is not captured by water quality testing at a single point in space and time. Where multiple piped or other-improved water source types were tested within a country, the percentages were computed as an average weighted by the population using each source type. The population receiving drinking water from each source in 2004 to 2005 was estimated from RADWQ project reports.

The RADWQ study made no attempt to assess the safety of water from unimproved sources, other than the case of tanker trucks in Nigeria [9]. In the absence of such data, we assumed that 100% of unimproved sources do not provide safe water. 

### 2.3. Synthetic Covariates from Principal Components Analysis

In order to estimate global access to safe water, empirical statistical models capturing the relationship between faecal contamination or sanitary risk proportions and country-level economic, governance, health, social and environmental characteristics were built. Covariates were chosen based on the availability of data for countries with 2010 JMP estimates and on their relation to drinking water quality. The following country-level indicators were explored: gross domestic product per capita (GDP) [16], the World Bank’s Government Effectiveness (GE) score [17], the Human Development Index [18], the Water Quality Index (WQI) from Yale’s Environmental Performance Index [19], annual aggregate precipitation [16], percent of population attaining tertiary education [20], and under-5 diarrheal morbidity rates [16]. Due to many of these variables being highly correlated, and the small size of our dataset, the models were limited to one or two covariates. Principal Components Analysis (PCA) was used to create uncorrelated synthetic variables that captured the most variance in those national characteristics. This analysis was performed with Stata 10.1.

### 2.4. Predicting Drinking Water Safety Proportions for Piped and Other-Improved Sources

Four fractional logit models were built based on the data points listed in Table 2, one for each of the proportions of: safe piped sources, safe other-improved sources, safe piped sources with elevated sanitary risk, and safe other-improved sources with elevated sanitary risk. We modeled the sanitary risk proportions of safe sources, since faecally-contaminated sources were assumed to be unsafe regardless of their level of sanitary risk. The fractional logit model as developed by Papke and Wooldridge [21], rather than minimizing the sum-of-squares error, is a quasi-maximum likelihood method. The fractional logit is superior to an ordinary least squares (OLS) regression because the predicted values of such models are not guaranteed to be restricted to values between 0 and 1. This method is also superior to the common alternative of performing an OLS regression on the logit transformation of the proportion (log-odds regression), in that the predicted values of the proportions from such a model are not recoverable without making significant assumptions.

Candidate covariates used in the models were the first three PCA components from the analysis described in section 2.3. For each of the four dependent variables, models using combinations of these three components were run and the models having the greatest log-likelihoods while maintaining a significant difference from the null model were chosen. 

**Table 2:** Cross-tabulation of TTC contamination and sanitary risk for piped and other-improved water sources [8,9,10,11,12].

Piped *					
	**% Sanitary risk category safe**	**TTC Count (cfu/100 mL)**			
		**<1**	**1–10**	**10–100**	**>100**
**Ethiopia**					
0–2 (Very Low San. Risk)	88%	78	1	8	2
3–5 (Low San. Risk)	89%	220	9	14	5
6–8 (Med. San. Risk)	70%	40	5	11	1
9–10 (High San. Risk)	0%	0	1	0	0
**Jordan**					
0–2 (Very Low San. Risk)	100%	1233	1	0	0
3–5 (Low San. Risk)	100%	404	0	0	0
6–8 (Med. San. Risk)	NA	0	0	0	0
9–10 (High San. Risk)	NA	0	0	0	0
**Nigeria**					
0–2 (Very Low San. Risk)	89%	108	10	3	1
3–5 (Low San. Risk)	79%	263	48	23	1
6–8 (Med. San. Risk)	72%	115	14	26	5
9–10 (High San. Risk)	23%	3	3	5	2
**Tajikistan**					
0–2 (Very Low San. Risk)	91%	1038	100	0	0
3–5 (Low San. Risk)	72%	91	35	1	0
6–8 (Med. San. Risk)	48%	10	11	0	0
9–10 (High San. Risk)	NA	0	0	0	0
Other-Improved **					
	**% Sanitary risk category safe**	**TTC Count (cfu/100 mL)**			
		**<1**	**1–10**	**10–100**	**>100**
**Ethiopia**					
0–2 (Very Low San. Risk)	94%	192	2	3	8
3–5 (Low San. Risk)	72%	567	76	94	46
6–8 (Med. San. Risk)	41%	122	46	76	53
9–10 (High San. Risk)	0%	0	2	0	3
**Nigeria**					
0–2 (Very Low San. Risk)	78%	364	71	31	0
3–5 (Low San. Risk)	80%	256	31	33	1
6–8 (Med. San. Risk)	81%	105	9	15	1
9–10 (High San. Risk)	38%	12	9	10	1
**Tajikistan**					
0–2 (Very Low San. Risk)	77%	121	28	6	2
3–5 (Low San. Risk)	89%	146	15	3	0
6–8 (Med. San. Risk)	54%	7	4	1	1
9–10 (High San. Risk)	NA	0	0	0	0

* All crosstabulations from RADWQ project reports; The Nicaragua report did not include them. ** No other-improved sources tested in Jordan.

The resulting models were used to extrapolate the proportions calculated from the RADWQ countries to the remaining countries with JMP data for 2010. The predicted proportion of piped or other-improved sources uncontaminated with TTC for each country was multiplied by the corresponding JMP estimate of the population using piped or other-improved water, respectively, to estimate the population with access to microbiologically safe piped or other-improved water in each country. Similarly, the predicted proportion of uncontaminated piped or other-improved sources with elevated sanitary risk was multiplied by the estimated population with access to uncontaminated piped and other-improved water sources. 95%-confidence interval upper and lower bounds for each figure were calculated by using the standard errors of the model coefficients. This method of calculating confidence intervals assumes the RADWQ studies are perfectly accurate in their reported percentages. This assumption is necessary as the complete RADWQ data sets were not available and we were therefore unable to calculate standard errors for the RADWQ country percentages.

### 2.5. Adjusting MDG Target 7c Progress Estimates

We re-estimated MDG progress towards global safe water access by accounting for faecal contamination of piped or other-improved water sources, as well as sanitary risks of piped or other-improved sources for which faecal contamination was not detected. The 1990 baseline estimates obtained from JMP were multiplied by the estimated proportions of people with piped and other improved water sources for which such sources were estimated to be unsafe by virtue of having tested positive for TTC. This was repeated to account for piped and other-improved sources with sanitary risks. In the absence of any trend data, proportions estimated based on the 2004–2005 RADWQ data were used to estimate both 2010 and 1990 water source safety. Projections of the global proportion of people without access to safe water in 2015 according to JMP data, and accounting for water quality, were based on linear extrapolations of the 1990–2010 JMP and water quality-adjusted data, respectively. This is the same method used by JMP to make its projections [22]. The 2015 MDG targets were calculated by halving the original and recalculated 1990 baselines. 

## 3. Results

The results of the RADWQ studies in the form of cross-tabulations of detected TTC contamination levels and sanitary risk scores, aggregated into piped and other-improved sources, are shown in Table 2. Within each country, TTC contamination rates for each water source type generally increase with increasing sanitary risk, indicating the expected association between water contamination and the presence of sanitary risk factors. 

The aggregated results of the RADWQ studies are presented in Table 3. The relationship between sanitary risk and TTC contamination differs between countries. For example, even though all Jordan’s piped water supplies are uncontaminated, almost a quarter have more than two sanitary risks; while less than 9% of Tajikistan’s uncontaminated piped supplies have more than two sanitary risks. 

Data were available such that the PCA could be performed for 150 countries, accounting for 92.5% of the global population (Table 4). The first three components explained 84% of the variance among these countries. The first component shows that GDP, GE, HDI, and Tertiary Education rates all covary and contribute similar information. The second component is dominated by annual aggregate precipitation, and the third component by the WQI. The spread of the five RADWQ countries with respect to the dominant variables of the first three components are shown in Table 5.

**Table 3:** Contamination of piped and other-improved sampled sources with TTC, and sanitary risk of safe sources [8,9,10,11,12].

Source type, by country	Thermotolerant coliform (TTC) contamination *		Sanitary risk of safe sources
	TTC-Uncontaminated (Safe) (%)	Sources sampled ( *n*)	>2 Sanitary Risks (%)
**Ethiopia**			
Piped	87.6	838	60.7
Other-Improved	55.0	764	67.1
**Jordan**			
Piped	99.9	1639	24.7
Other-Improved	NA	0	NA
**Nicaragua**			
Piped	85.5	600	47.3
Other-Improved	33.5	888	56.5
**Nigeria**			
Piped	77.0	630	77.9
Other-Improved	76.0	949	50.6
**Tajikistan**			
Piped	88.6	1286	8.9
Other-Improved **	82.0	334	55.8

* Data aggregated from RADWQ reports. TTC contamination judged against guideline value of <1 TTC per 100 mL as indicated in the WHO Guidelines for Drinking Water Quality [15]; ** Only 44% of the protected springs in Tajikistan were found to be adequately protected for designation as an improved source.

**Table 4:** Factor loadings, Eigenvalues, and Variances accounted for from PCA.

Variable	Comp 1	Comp 2	Comp 3	Comp 4	Comp 5	Comp 6	Comp 7
*Eigenvalue*	4.19	1.06	0.623	0.476	0.326	0.180	0.138
pc GDP	0.422	0.121	0.353	0.204	−0.429	0.647	0.199
GE	0.422	0.151	0.336	0.348	−0.0785	−0.715	0.219
HDI	0.441	0.0920	0.258	−0.101	0.399	0.0799	−0.745
Precipitation	−0.102	0.913	0.0051	−0.380	0.0029	−0.0178	0.106
WQI	0.331	0.237	−0.793	0.433	−0.0363	0.0497	−0.123
U5 Diarrhea	−0.384	0.214	0.227	0.642	0.537	0.210	0.100
Tert. Educ.	0.427	−0.137	−0.127	−0.280	0.601	0.131	0.567
*Proportion*	0.599	0.152	0.0885	0.0679	0.0466	0.0258	0.0197
*Cumulative*	0.599	0.751	0.839	0.908	0.955	0.980	1.00

**Table 5:** Spread of countries on dominant PCA Covariates.

	GE	Precipitation (mm)	WQI
Ethiopia	−0.35	848	55.3
Jordan	0.08	111	11.9
Nicaragua	−0.96	2391	57.1
Nigeria	−1.2	1150	20.1
Tajikistan	−0.91	691	42.8

We used the fractional logit models shown in Table 6 to predict proportions of piped and other-improved sources that provide safe water in every country for which 2010 JMP estimates were available. A comparison of the RADWQ countries’ measured and predicted proportions is shown in Table 7. We estimate that 1 billion (confidence interval 0.75 to 1.6 billion) of the 5.8 billion using piped or other-improved sources receive faecally-contaminated water. This lowers the number of people estimated to use safe water from 5.8 billion (the 2010 JMP figure) to 4.8 billion, and increases the number or people with unsafe water from 0.78 billion to 1.8 billion as of 2010. 

**Table 6:** Fractional Logit Models of Water Source Safety Proportions.

	Model			
Variable	% Piped Sources Safe	% Other-Improved Sources Safe	% Safe Piped Sources w/Elevated Sanitary Risk	% Safe Oth. Imp. Sources w/Elevated Sanitary Risk
Comp 1	0.879 **		−1.131 ***	
	(0.286)		(0.285)	
Comp 2	−0.436 *	−0.792 ***		
	(0.209)	(0.159)		
Comp 3				−0.350 ***
				(0.099)
Constant	3.188	0.370	−1.819	0.345
	(0.566)	(0.236)	(0.707)	(0.075)
N	5	4	5	4
Residual df	2	2	3	2
Log pseudo-likelihood	−1.2305	−1.65	−2.119	−1.773

* p < 0.05; ** p < 0.01; *** p < 0.001; one-tailed tests for hypothesized effects, robust standard errors in parentheses.

Of these 4.8 billion using safe water, approximately 1.2 billion people (confidence interval 0.75 to 2.1 billion) receive water from sources that are at risk of faecal contamination by virtue of having greater than two of the common sanitary risks for that source type as defined by RADWQ [14]. If a more stringent definition of safety (requiring both no faecal contamination and low sanitary risk) is used, then the estimate of the number of people with unsafe water is 3 billion, (confidence interval 1.5 billion to 3.9 billion). 302 million people reside in countries for which JMP estimates do not exist. An additional 370 million people reside in countries for which JMP estimates exist, but for which the data necessary for principal components analysis was unavailable. The safety of the water received by these 370 million is not estimated.

**Table 7:** Comparison of Measured and Predicted Proportions.

	% Piped Sources Safe		% Other-Improved Sources Safe		% Safe Piped Sources w/Elevated Sanitary Risk		% Safe Oth. Imp. Sources w/Elevated Sanitary Risk	
**Country**	*RADWQ*	*Predicted*	*RADWQ*	*Predicted*	*RADWQ*	*Predicted*	*RADWQ*	*Predicted*
Ethiopia	88%	85%	55%	55%	61%	59%	67%	57%
Jordan	100%	98%	NA	87%	25%	18%	NA	48%
Nicaragua	86%	85%	34%	33%	47%	33%	57%	56%
Nigeria	77%	77%	76%	68%	78%	73%	51%	51%
Tajikistan	88%	93%	82%	76%	9%	18%	56%	55%

**Figure 1:**
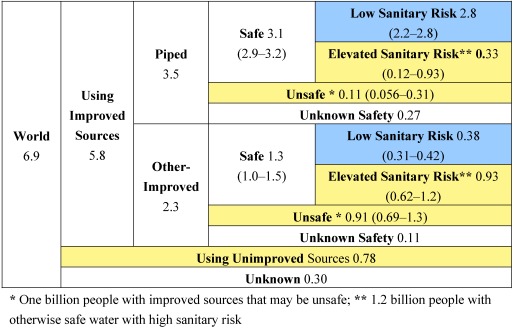
World population by water contamination status and sanitary risk for 2010 (billions).

The full categorization of the world population of piped and other-improved water source users into categories based on faecal contamination and sanitary risk using our estimation methods is shown in Figure 1, where upper and lower bounds are shown in parentheses where available. The world’s population is divided into those using improved sources, unimproved sources, and sources of unknown type. The improved source population is divided into those using piped and other-improved sources. Each of these two populations is divided into those using sources that are safe, unsafe, or unestimated safety. The populations using safe piped and safe other-improved sources are divided into those using sources with low and elevated levels of sanitary risks.

**Figure 2:**
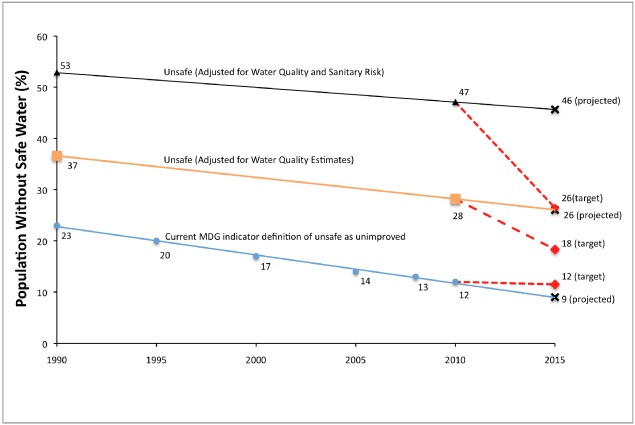
Comparison of MDG Target 7c baseline and target when including and excluding faecal contamination and sanitary risk in water safety.

Percentages of the world population for the purposes of adjusting the MDG Target 7c progress are calculated by assuming the populations with unknown water source types and water source safety are distributed similarly to the rest of the world. Accounting for faecal contamination based on our models increases the 1990 baseline estimate of the population without access to a safe water source from 23% to 37% (see Figure 2). In order to meet MDG Target 7c, the proportion without access to safe water would need to be reduced to 18% by 2015, while trends indicate this figure will be 26%. For 2010, the shortfall is 10 percentage points (680 million people), and we project an 8-percentage point shortfall in 2015, while JMP estimates, based on the indicator of use of an improved source, indicate that the MDG target has already been met. 

Using a more stringent definition of water safety that requires water sources be both uncontaminated and of low sanitary risk would result in a 1990 baseline of 53% of the population, a target of 26%, and a 2015 projection of 46%. This would result in a 21-percentage point shortfall in 2010 and a 20-percentage point shortfall in 2015.

## 4. Discussion

Our estimates of the population with access to safe water were based on several assumptions that could contribute to over- or under-estimation.

We used the >1 cfu/100 mL TTC level as a threshold for considering water microbiologically safe, as did the RADWQ studies. *E. coli* and *Enterrococci* have been shown to be better indicators of waterborne disease risks than total TTC [23]. This could result in an overestimate of the population actually at risk of waterborne disease, as some sources testing positive for TTC may not be positive for *E. coli*.

We assumed that the relationships between water quality and national characteristics described by the models in Table 3 hold for countries other than the five countries assessed by RADWQ. The causal factors determining water quality for a given source type may differ from country to country and the causal relationships determining water quality for a given source type may differ from country to country. In addition, countries differ substantially regarding the mixture of predominant source types. For instance, protected springs were the only non-piped improved water source assessed by RADWQ in Tajikistan, while boreholes and protected wells were assessed in Ethiopia, Nicaragua, and Nigeria [8,9,10,11,12]. All non-piped “improved” sources were aggregated and treated as “other-improved”. Given these country-to-country differences, the direction of error will differ from country to country although the overall effect on the global estimates may be relatively small as the errors are aggregated.

The sanitary risk estimates are based on the proportion of safe sources assessed as having greater than two of the sanitary risks listed as possible on the RADWQ survey forms. Moreover, the RADWQ project reports stated that the lists of sanitary risks used in the survey could have been better tailored to country conditions [8,9,10,11,12]. Each of the possible risks on these forms probably contributes to a different level of actual risk for water contamination, but are weighted equally in the RADWQ methodology [8,9,10,11,12,14]. The relationship between the number of sanitary risks and the prevalence and degree of water contamination differs between countries (Table 2). Since any single sanitary risk is significant, use of this threshold (three or more risk factors) is likely to underestimate the population using unsafe water. 

We assumed that no contamination occurs between the water point and the point and time of use. However, such contamination is known to occur [5]. Water that is safe in a distribution system at one point may become contaminated at another point before it is received by the user, due to deficiencies in the distribution system [24]. Additionally, water that must be transported manually from the source to the home, and any water stored in the home, as is common with other-improved sources, can become contaminated due to unsanitary storage conditions [25]. We did not account for this due to lack of representative data. The effect may be greater for the estimates for “other-improved” sources than for piped sources. However, the effect is likely to be significant in the case of piped sources with discontinuous service that encourage users to store water in the home. These assumptions would tend to lead to an underestimate of the population using unsafe water. 

We assumed that the proportion of piped and other-improved sources that provide safe water found in the RADWQ reports represent the safety of those sources over an entire year. This is likely incorrect, due to the transient nature of many contamination events [26]. One round of water quality testing is unlikely to capture the true extent of microbial contamination that might occur over a long period of time at a given source. As such, the RADWQ data likely substantively underestimate the proportion of water sources that are contaminated over an extended time period. The effect would be to underestimate the population using unsafe water.

We did not consider contamination from nitrates, arsenic, fluoride, or any of the chemical contaminants not tested for by the RADWQ project. This is because waterborne pathogens from faecal sources cause more disease than any other waterborne contaminant, and thermotolerant coliform bacteria measurement serves as an indicator for the presence of faecal contamination [15]. There is likely not complete overlap in terms of the types of contamination a given water source might face, so more water sources are probably unsafe than reliance on microbiological indicators alone would indicate. The effect of these assumptions is to underestimate the population using unsafe water.

We assumed that the proportion of piped and other-improved sources that are faecally contaminated, and the proportion of these with elevated sanitary risk, remained constant across time, at least backwards to 1990 for the purposes of recalculating the MDG baseline, and forwards to 2015 for projected proportions. This assumption was necessary due to a lack of equivalent data for the relevant time periods. The effect of this assumption will vary from country to country, and the overall effect on the global estimates may be relatively small as the errors are aggregated.

Since the RADWQ data was not disaggregated by rural and urban settings, we assumed that the proportions of sampled water sources complying with WHO guidelines and having significant levels of sanitary risk were the same in urban and rural settings. The effect of this assumption will vary from country to country.

We assumed that 100% of unimproved sources do not provide safe water. This is based on a lack of water quality data and a consequent reliance on the same methodology as JMP, treating all such sources as unsafe due to the lack of sustainability of access and lack of protection against contamination characteristic of such sources. This assumption could result in an overestimate of the number of people receiving faecally contaminated water. However, this assumption does not overestimate those receiving water from sources with elevated sanitary risks, since unimproved sources by definition lack sanitary protection. In addition, the MDG indicator accounts for both access and water quality [1]. Since unimproved sources may not meet the access criterion of the indicator, the potential error of the assumption is further reduced.

We did not account for household water treatment (HWT). Use of adequate HWT strategies in households can reduce the health risks of water from contaminated sources (both improved and unimproved sources) and also the risk arising from water contamination during transport and household storage [25,27]. Among a sample of 67 low and middle-income countries, using data from the nationally representative Demographic and Health Surveys (DHS), 1.1 billion people use HWT [27]. However, the DHS surveys used did not confirm responses indicating HWT use with physical indicators in the home of such use. Additionally, it is unknown how these people might be distributed over the actual quality of their source water, how many of these people only use water that they have treated, or to what degree these people sustain the HWT strategy for long periods of time. There also exists the risk of recontamination of water post-treatment [28]. The number of people relying on HWT receiving safe water is likely much smaller than 1.1 billion. As such, the effect of the assumption is likely to be a relatively small overestimate of the population using unsafe water.

The effects of the above assumptions will vary between countries. As such, the country-level estimates for water source compliance and sanitary risk are not presented, as these are likely to be imprecise. However, the overall effect on the global estimates may be relatively small as the errors are aggregated.

The confidence intervals were calculated using the standard errors of the model parameter estimates, and did not account for the standard error of the dependent variable. The standard errors of the reported RADWQ figures were unavailable. This results in an underestimate of the upper bounds and an overestimate of the lower bounds. 

## 5. Conclusions

We show that the MDG indicator (proportion of the population using an improved water source) used in assessing progress towards MDG Target 7c results in a substantive underestimate of the proportion of the population using unsafe water. The 2010 JMP data shows that 780 million people (11% of the population) use unimproved sources. We estimate that 1 billion (between a lower bound of 750 million and an upper bound of 1.6 billion) people using piped or other-improved water sources receive unsafe water, meaning 1.8 billion people did not have access to safe water in 2010. 

Using a definition for safe water that includes the absence of faecal contamination in a one-off sample from a piped or other-improved source shows that the current indicator, based on the definition of an improved source alone, underestimated the progress required to meet the drinking-water component of MDG Target 7c by 10% of the global population, whereas the 2010 JMP progress estimate suggests that the MDG drinking-water target has already been achieved.

We estimate that an additional 1.2 billion (between 750 million and 2.1 billion) people using safe piped or other-improved sources are using sources that are at elevated risk of contamination unlikely to be detected by one-off, or perhaps even routine monitoring. 

While these estimates are imprecise, their magnitude and health and development implications suggest that greater attention is needed to better understand and manage the problem of contamination of improved water sources.

This study highlights the substantive differences between population estimates using water from an improved source and estimates of populations using water free of faecal contamination and water with low risk of faecal contamination. As increasing populations use piped and other-improved water sources it is likely that increasing attention will be paid to water safety. The demand for evidence to inform effective policy making will increase in parallel. Here we use data from large scale dedicated surveys to gain preliminary insights. While the RADWQ methodology has provided valuable information regarding national water safety in terms of contamination and sanitary risk factors, further work is required to identify the most appropriate means to secure greater and more targeted evidence to inform decision-making.
